# Role and mechanism of fibroblast-activated protein-α expression on the surface of fibroblast-like synoviocytes in rheumatoid arthritis

**DOI:** 10.3389/fimmu.2023.1135384

**Published:** 2023-03-17

**Authors:** Zihan Wang, Jinping Wang, Tianyi Lan, Liubo Zhang, Zeran Yan, Nan Zhang, Yuan Xu, Qingwen Tao

**Affiliations:** ^1^ Traditional Chinese Medicine Department of Rheumatism, China-Japan Friendship Hospital, Beijing, China; ^2^ Graduate school, Beijing University of Chinese Medicine, Beijing, China

**Keywords:** rheumatoid arthritis, fibroblast-activated protein-α, fibroblast-like synoviocytes, cell function, targeted therapy

## Abstract

Fibroblast-activated protein-α (FAP) is a type II integrated serine protease expressed by activated fibroblasts during fibrosis or inflammation. Fibroblast-like synoviocytes (FLSs) in rheumatoid arthritis (RA) synovial sites abundantly and stably overexpress FAP and play important roles in regulating the cellular immune, inflammatory, invasion, migration, proliferation, and angiogenesis responses in the synovial region. Overexpression of FAP is regulated by the initial inflammatory microenvironment of the disease and epigenetic signaling, which promotes RA development by regulating FLSs or affecting the signaling cross-linking FLSs with other cells at the local synovium and inflammatory stimulation. At present, several treatment options targeting FAP are in the process of development. This review discusses the basic features of FAP expressed on the surface of FLSs and its role in RA pathophysiology and advances in targeted therapies.

## 1 Introduction

Rheumatoid arthritis (RA) is an aggressive immune-mediated disease (1) with a worldwide prevalence of about 0.46% (2). Although the specific causes of RA occurrence remain unknown, genetic and environmental factors may contribute to the onset of the disease. Autoimmune reactions that occur before clinical symptoms are identified as signs of the disease onset and are also thought to be triggers of RA (3). Synovitis is the predominant pathological change in RA, leading to irreversible joint bone destruction in the later stages (4). The cross-linking reaction between immune cells and inflammatory cells in the local synovial region is a constant concern in RA.

Fibroblast-like synoviocytes (FLSs) (5) are generally considered to be widely distributed in the synovium and play an important role in RA. As intrinsic mesenchymal cells in synovial structures, FLSs play an important role in maintaining the dynamic balance of the internal environment. They possess the characteristics of inflammatory cells and are also considered a key pro-inflammatory factor. Although targeted modulation of FLS function has become one of the new directions in RA treatment (6), the actual ability of FLS targeting to induce RA remission remains to be clinically validated. However, this does not undermine the therapeutic potential of FLSs. Importantly, promising therapeutic surface biomarkers are yet to be fully understood, which is the primary limiting factor for the practical application of FLS-targeting therapy.

Fibroblast-activated protein-α (FAP)—a serine protease expressed on the FLS surface (7)—is a surface biomarker that is barely expressed in normal adult FLSs and is associated with FLS activation. FAP is only highly expressed in pathological tissues, including RA-lesioned synovial tissues and various tumor stromal tissues. The regulatory effects of FAP in RA have been well demonstrated. For example, targeting the depletion of FAP^+^ FLSs has been shown to slow the development of arthritis in mice (8). These exciting discoveries have allowed FAP to emerge as a high-profile research biomarker in recent years. The present review focuses on FAP-mediated functional phenotypic changes that occur in FLSs and the related regulatory mechanisms of FAP expression, which may deepen our understanding of RA pathogenesis and provide clues for identifying new therapeutic targets for RA.

## 2 Sources and surface markers of FLSs

The synovium is divided anatomically into the lining and sublining layers. The lining layer connects to the joint cavity, and the sublining layer consists of a connective tissue network of sparse cells and blood vessels (9). The synovial tissue mainly consists of a class of cells with stem cell-like characteristics and immunomodulatory capacity, which have previously been described as mesenchymal stem cells (MSCs), FLSs, synovial fibroblasts, type B synoviocytes, etc. The term “FLS” is often preferred to represent these synovial-derived cells in RA studies. Although these complex terms are used interchangeably, the different designations represent different stages of cell growth and MSCs may represent immature FLSs (10).

FLSs are derived from synovial tissue, and these spindle-like cells have strong differentiation potential, contributing to tissue separation, forming functional joint cavities, exhibiting positive cartilage formation and osteogenesis, and exerting immunomodulatory effects through intercellular contact and secretion of cytokines, which are important for the physiological development of the synovium and joints (11). In pathological situations, FLSs are the main effector cells leading to exacerbation of RA synovitis and bone erosion, undergoing sustained proliferation and migration induced by local injury signals (12).

**Figure 1:**
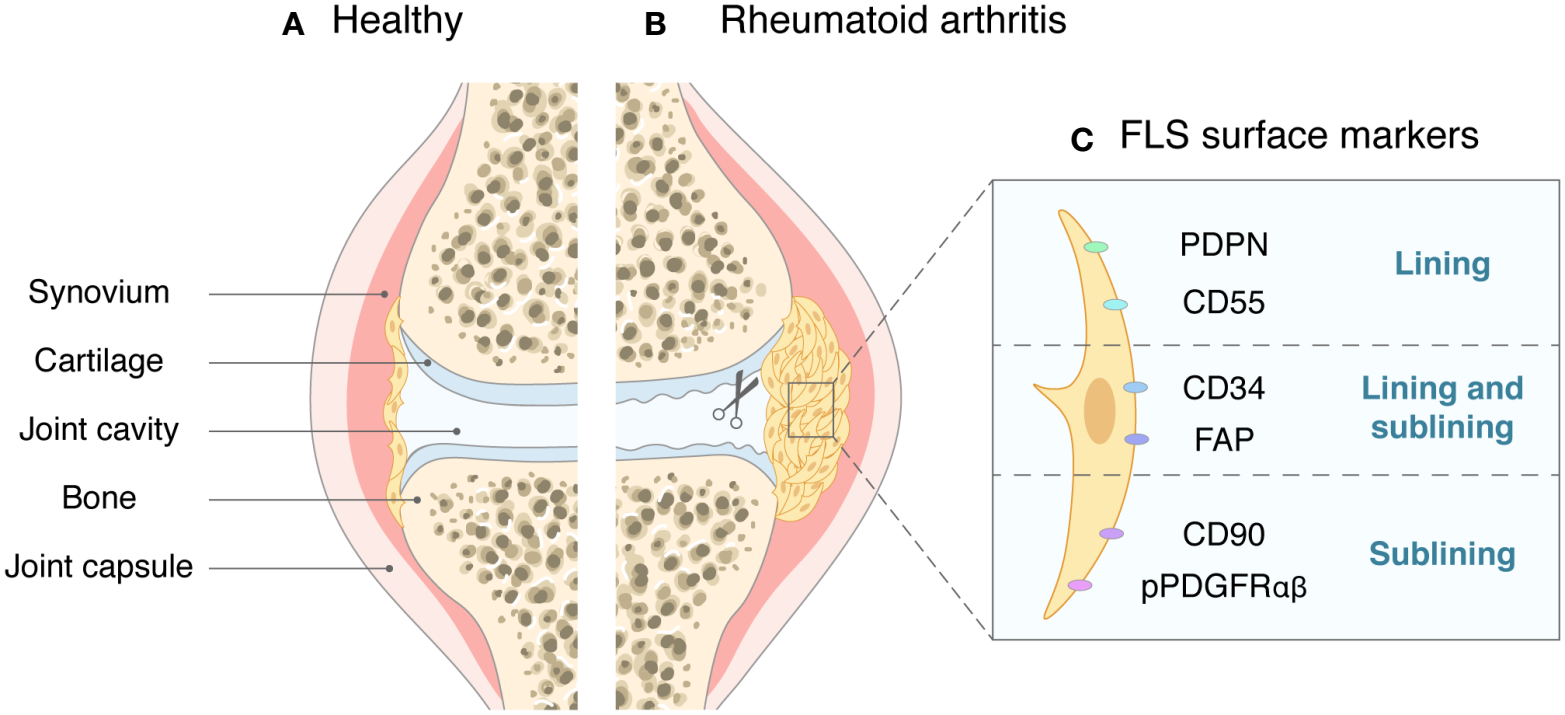
The synovial joint in health and in RA. **(A)** In healthy joints, the synovial tissue is sparse, with only one or two layers of cells. **(B)** In RA, synovial cells proliferate and become invasive, causing cartilage and bone to erode. **(C)** Surface markers of FLSs and their main locations.

FLSs are found in both the lining and sublining layers and can also be detected in the inflammatory synovial exudate in RA patients (13). FLSs distributed in different locations of the synovial tissue differ in subtype classification, and the expression patterns of some surface biomarker proteins such as CD90 (Thy1), CD34, and CD55 can indicate the functions and locations of FLSs (14). These surface markers are expressed in the context of FLS activation. The most frequently mentioned CD90^-^ FLSs are mainly present in the lining layer and can express cytokines such as matrix metalloproteinases (MMPs) and receptor activator of nuclear factor kappa-B ligand (RANKL) to induce bone destruction. In contrast, CD90^+^ FLSs are mainly present in the sublining layer and are more inclined to participate in inflammatory responses (15). Podoplanin (PDPN) is a transmembrane glycoprotein on the surface of FLSs, and activated PDPN^+^ FLSs are mainly located in the synovial lining layer (16). A recent prospective cohort study suggested that activated PDPN is associated with the development of RA in individuals at high risk of the disease (17). Another transmembrane phosphorylated protein, CD34, is widely distributed on the FLS surface in the lining and sublining layers. The CD34^+^ FLS subpopulation has been reported to be more invasive and migratory *in vitro*, releasing higher levels of inflammatory factors after stimulation with tumor necrosis factor (TNF) (18). CD55 is a cell surface protein associated with activation of the complement system and was previously thought to be closely associated with peripheral blood cells and cancer cells (19, 20). Recent studies have identified CD55^+^ FLSs, a subpopulation distributed in the synovial lining layer and associated with endothelial cell proliferation and reactive oxygen species-regulated responses (21, 22). Moreover, the cell adhesion factor cadherin-11 (CDH-11) and phosphorylated platelet-derived growth factor receptor (pPDGFRαβ) are known to be expressed throughout the synovial layers. In addition, pPDGFRαβ^+^ CDH11^-^ FLSs are specifically distributed in the sublining layer. This subpopulation of FLSs is thought to possess resistance to cell death and show greater involvement in inflammation (23). With advances in research methods, FLS classification can be better determined by combining single-cell sequencing, high-throughput sequencing, and RNA-seq analysis (24, 25), and defining FLS subgroups on the basis of differences in surface biomarker expression can help identify the different locations and functional roles of FLSs (**Figure 1**).

The transmembrane protein FAP is present on the surface of all FLSs, and its activation correlates with the degree of inflammation and bone destruction in RA joints (26). FAP, together with dipeptidyl peptidase (DPP)-2/4/6/8/9/10, especially DPP-4, form the post-prolyl peptidase family (27), with 50%-70% sequence identity between the two structural domain sequences (28). This structure shows that FAP has a unique function. Overexpression of FAP is often closely associated with abnormal activation of FLSs, in which phenotypic characteristic activities such as proliferation, invasion, inflammation, and immunity of tissue cells in RA synovial sites occur, contributing significantly to the progression of RA.

## 3 Plasticity of FAP expression

### 3.1 Activation by immune and inflammatory response

Although FLSs are involved in the regulation of immune homeostasis in healthy synovial membranes under physiological conditions, they are aberrantly activated in the abnormal immune and inflammatory environment of RA, causing the expression of the associated pathogenic phenotype (29). For example, T cell chemokines can stimulate FLS activation, maintain the inflammatory phenotype of the cells, and use FLSs as antigen-presenting cells, leading to T cell activation and proliferation that act synergistically with the abnormal immune system to further drive inflammation (30, 31). However, the persistent inflammatory secretion of FLSs disappears upon removal of these cellular stimuli, while some of the cell surface biomarkers remain activated in the legacy (32, 33). Persistently high expression of surface proteins has a memorability, which makes FLSs more sensitive to inflammatory stimuli. RA begins with local inflammation, and this inflammatory environment activates key cell surface proteins that inflict the pro-inflammatory phenotype of FLSs, exacerbating local inflammation through a feedback loop to the point where the synovial tissue undergoes a waterfall cascade response at a later stage and spreads inflammation to other parts of the body.

Multiple studies have reported that inflammation can induce FAP. Early research in the field of atherosclerosis proved that macrophage-derived tumor necrosis factor-α (TNF-α) targets the structural domain of FAP, resulting in dose-dependent upregulation of FAP expression (34), while several *in vitro* experiments in myocardial infarction and oncology have demonstrated that FAP can be induced by transforming growth factor-β (TGF-β) through the classical drosophila mothers against decapentaplegic protein (SMAD) family member 2/3 pathway (35, 36). Another study proposed that active FAP on chondrocyte membranes in patients with osteoarthritis may be activated after stimulation with pro-inflammatory cytokine interleukin (IL)-1 and oncostatin M (37). These findings underpin the important regulatory role of inflammatory signaling in FAP expression.

FAP can be non-enzymatic in subsequent signaling by binding to specific receptors, such as αvβ6-integrin. This process has been shown to promote TGF-β secretion and influence the cellular inflammatory response (38). In terms of its biological structure, FAP exists as a 170-kDa protein on the surface of FLSs and can be activated only when it is assembled as a dimer under specific conditions by adjusting the structural domain (e.g., binding DPP-4) based on its spatial structure (39). N-linked glycosylation modification is also a part of the processing to form a functional FAP (40). Related oncological studies have consistently reported that the highly inflammatory environment drives FAP activation and participates in processes related to FAP dimer assembly, receptor binding, and glycosylation modification.

As an inflammatory and immune disease, RA is recognized to show a clear association with FAP, and we hypothesize that a similar initiation process occurs in the local synovial microenvironment. However, more experimental evidence is needed to explain the causal relationships among FAP and immune and inflammatory responses.

### 3.2 Epigenetic regulation

As a protein located on the cell surface, the functions of FAP can be controlled at the transcriptional level; therefore, identifying such transcriptional regulators of FAP mRNA may reveal the epigenetic regulatory mechanism of FAP. The transcription factor early growth response-1 (EGR-1) is mainly involved in the regulation of tissue injury, immune response, and fibrosis (41), and its high expression has been linked to the progression of the RA inflammatory response (42) and can regulate RA-FLS proliferation and apoptosis through the extracellular regulated protein kinases (ERK) signaling pathway (43). Zhang JP et al. identified the FAP promoter in human and mouse embryo fibroblasts and found that the EGR-1 binding site with a 2-kb promoter fragment was required for FAP expression and that the promoter could only be transactivated in FAP^+^ cells (44). These results suggest that EGR-1 may play a role in the activation of RA-FLS by binding to FAP. Such potential connections are worth further exploration in the field of RA research. Notably, downregulation of EGR-1 only partially blocked FAP transcription, suggesting that transcription factors that bind other sites are also involved in the regulation of FAP mRNA transcription.

TGF-β1, a multifunctional key biomediator, is highly expressed in RA synovial tissue and affects alterations in the 28-joint disease activity score and erythrocyte sedimentation rate (ESR) in RA patients (45). It has been also linked to increased expression of multiple inflammatory factors (46). One study conducted in cardiac myocytes suggests that the TGF-β superfamily can activate inflammation through the classical SMAD signaling pathway to promote FAP expression (35). In addition, TGF-β1 has been shown to be involved in FAP transcription and can act as one of the regulators of the FAP promoter in the glioblastoma microenvironment (47). Through luciferase labeling, Li WL et al. found that TGF-β1 could interact with another 5.4-kb FAP promoter region and that TGF-β1 could promote the activation of FAP on the surface of fibroblasts in mice models of fibrotic disease (48). Collectively, these findings confirmed the additional role of traditional bioregulatory factors in FAP transcription as well as their pro-inflammatory effects. These studies expand our understanding of the complex network underlying FAP functional regulation. However, the application of this understanding in the context of the pathological mechanisms of RA remains an important question.

Several splice variants have been reported to appear in the translation of FAP from mRNA to protein, but it remains unclear whether the resulting protein products are all biologically active. Three enzymatically active FAP splice variants with similar structure and expression were identified in immunodeficient mouse embryonic tissue. However, they all lacked the extracellular chain near the transmembrane region, resulting in the loss of certain FAP functions (49). The evidence has indicated that different splice sites of FAP mRNA may also affect the function, but the reasons for these differences require further research at the epigenetic level.

Genetic modifications of RNA have also been shown to affect FAP expression. MicroRNA (miRNA) is a conserved small non-coding RNA that regulates post-transcriptional gene expression (50). Several miRNAs, such as miR-30a and miR-204, have been found to be negatively correlated with FAP expression and were not only involved in the induction of FAP expression by TGF-β1 in interstitial pulmonary fibrosis (51), but were also verified to target and inhibit FAP directly in oncological studies (52, 53). Meanwhile, increased expression of miR-630, miR-200c, and miR-155-5p resulted in the upregulation of FAP levels in some tumor diseases (54–56). Long non-coding RNAs (lncRNAs), another class of RNA molecules that do not encode a protein, have been considered to be regulators of FAP. For instance, bioinformatics analysis revealed that lncRNA AC009099 was positively correlated with FAP expression and may be regulated from the AC009099/miR-7152/FAP pathway in hepatocellular carcinoma (57), while lncRNA HIPK1-AS has shown similar effects in cervical cancer (58). Several studies from the field of oncology have demonstrated that exosomes promote FAP expression and that lncRNA Gm26809 and LINC00355 play essential roles in this process by recoding for fibroblasts (59, 60). The mechanisms by which non-coding RNAs regulate FAP are not yet well-studied, especially in relation to RA. Thus, additional studies are required to identify the transcriptional regulators and reveal evidence for the epigenetic and biomechanical regulation that will promote the development of FAP-targeted therapy.

## 4 Functions of FAP-mediated FLSs

**Figure 2:**
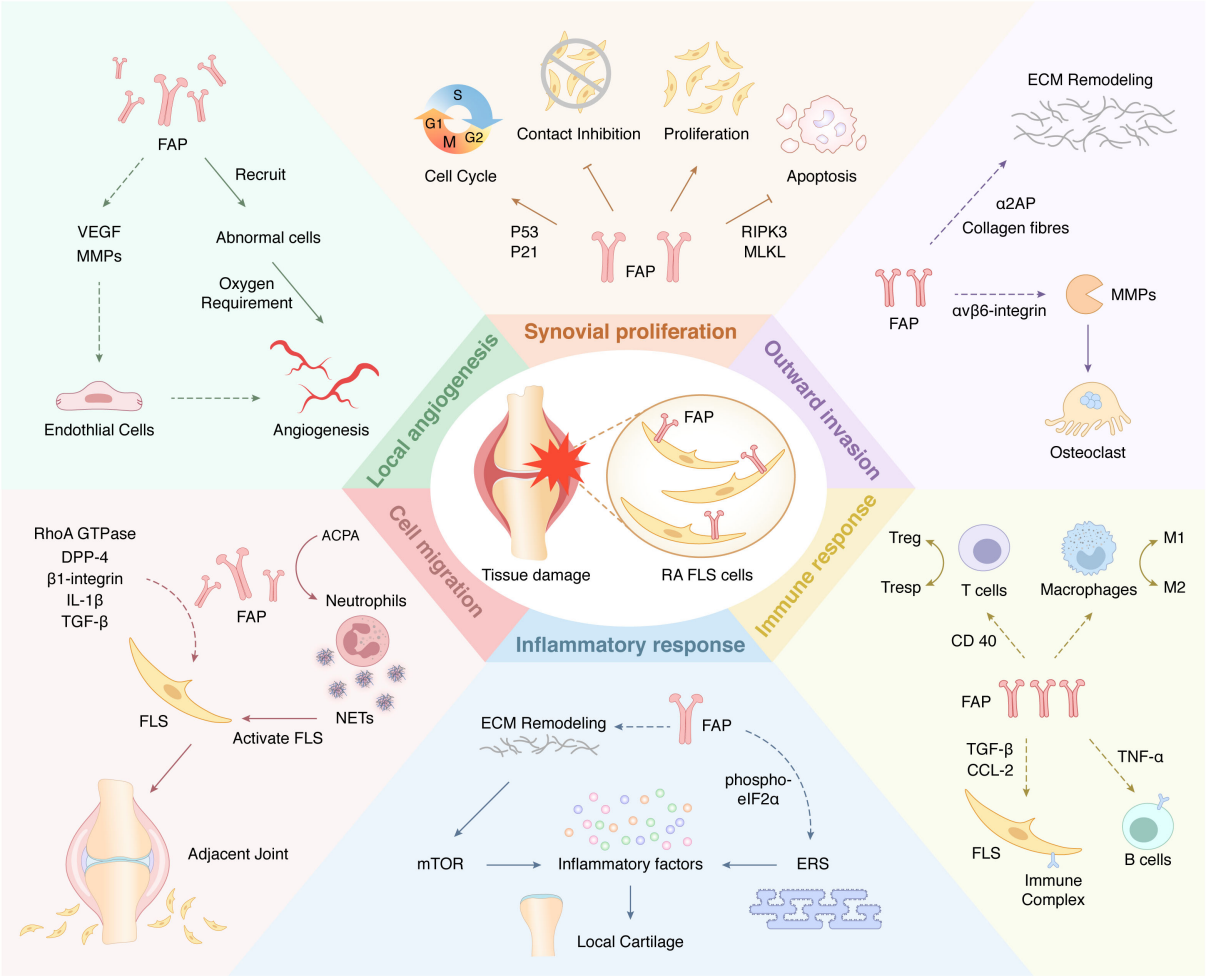
A Schematic view of cellular functions of FAP-mediated FLSs in RA. Arrows (↓) indicate positive impact, while inverted Ts (⊥) indicate negative impact. The dotted arrows (—) indicate studies that exist but have not yet been validated in the RA field.

FAP is always expressed on the surface of fibroblasts associated with various diseases (61–63). This protein plays a unique role in most physiological and pathological processes and has previously received considerable attention in areas such as cancer and heart disease. However, FAP is not an essential protein for life activity. A previous study of mice with myocardial infarction found that FAP knockout did not result in significant developmental defects (64). Therefore, FAP overexpression may play a more crucial role in pathological activities. The role of FAP in RA has received increasing attention in recent years (65). As one of the surface markers of FLSs, FAP is always overexpressed in RA-FLS (66), which co-acts with other cells and proteins to confer FLSs with the corresponding function and promote RA development (**Table 1**; **Figure 2**).

**Table 1:** Correlation factors, targets and biological responses associated with FAP-mediated FLSs functions.

Functions	Correlation Factors	Targets	Biological Responses	Fields of study	*Ref.*
**Immune response**	CD40	T cells	Initiate T cell action, provide epitopes and binds receptors, regulate phenotypic transformation	RA, Oncology	(67, 68, 69, 70, 71)
	TNF-α	B cells	Regulate the degree of immune response	RA, Influenza	(72, 73)
	TGF-β, CCL-2	FLSs	Amplify the immune response	Oncology	(74, 75)
		Macrophages	Promote inflammatory response, regulate phenotypic transformation	Atherosclerosis, Oncology	(76, 77)
**Inflammatory response**	inflammatory factors	cartilage	Promote cartilage inflammation	RA	(78)
	phospho-eIF2α	ERS	Imbalanced ERS produces high levels of cytokines	Dermatology	(79)
	mTOR	ECM	Alter lipid metabolism and collagen deposition	Oncology, IPF	(80, 81)
**Outward invasion**	collagen fibres, α2AP	ECM	ECM remodeling	Oncology	(82, 83, 84)
	αvβ6-integrin, MMPs		Promote cell invasion	Oncology	(38)
**Cell migration**	IL-1β, TGF-β, RhoA GTPase, DPP-4, β1-integrin	FLSs, MSCs	Constitute cell pseudopods and promote differentiation of MSCs to FLSs	Oncology	(85, 86, 87, 88)
	ACPA	Neutrophils, NETs	Activate FLSs	RA	(89, 90)
**Synovial proliferation**	P53, P21	FLSs	Regulate the cell cycle and relieve contact inhibition	RA	(66)
	RIPK3, MLKL	Neutrophils	Inhibit necrotic apoptosis	RA	(91)
**Local angiogenesis**		local abnormal cells	Generate blood vessels to meet oxygen and nutrient requirements	RA	(92)
	VEGF, MMP-1, MMP-9	endothelial cells	Endothelial cell proliferation promotes angiogenesis	Oncology, Corneal stroma, Adipose Tissue	(93, 94, 95, 96)

### 4.1 Immune response

FLSs can contribute to synovial immunity by directly secreting cytokines to activate T cells and recruit relevant immune cells, including lymphocytes and macrophages, to reach the inflamed tissue, resulting in a local infiltrative state (97, 98). The extracellular structural domain of FAP includes an eight-bladed β-propeller domain, which is thought to provide an abundant T cell epitope (67) to participate in T cell-mediated cellular immune processes. FLSs are also used as an immunosurveillance regulator in cancer and show a rich immune cross-linking effect with T cells, for instance, when CD4^+^ T cells are co-cultured with FLSs, which promotes the adhesion of monocytes (68). Meanwhile, RA-FLSs can bind to T cell receptors to stimulate T cell differentiation and act as an antigen-presenting cells for T cells (69), extracting and presenting autoantigens and human type II collagen to antigen-specific T cells. FLSs in inflamed synovial tissues release numerous chemokines, including CD13, IL-21, and IL-27 (99–101), which can act as chemoattractants for T cells, and the TGF-β/SMAD pathway is thought to be involved in the cross-linking between FLSs and immune cells in RA (102). RA-FLSs can show direct intercellular contact with T cells (103), promoting T cell activation and infiltration. One study reported that activated RA-FLSs can play a dual role between regulatory T cells (Treg) and responder T cells (Tresp), shifting the original homeostasis toward a pro-inflammatory state (104). In addition, FAP has been shown to act as an activator of CD40 in tumor models of mice, enhancing dendritic cell activation and initiating T cell action (70). FAP has been speculated to participate as an important surface marker in the interaction of FLS with T cells.

B cells participate in the pathogenesis of RA by producing autoantibodies and can act as an efficient class of antigen-presenting cells (105). RA-FLSs can express a series of B cell nutrients such as B lymphocyte stimulator and vascular cell adhesion molecule-1 in enhancing intercellular adhesion (106), while overexpression of hypoxia-inducible factor-1α (HIF-1α) promotes the upregulation of the cell contact mediator IL-15 on the RA-FLS surface (107), resulting in prolonged B cell survival and a reduced apoptosis rate. B cells can overexpress TNF-α, which induces RA-FLS activation through the ERK1/2 and Janus kinase/signal transducer and activator of transcription (JAK/STAT) signaling pathways (72). A study on the influenza A virus showed that FAP is involved in a series of interactions between fibroblastic reticular cells and B cells, and FAP depletion leads to the loss of B cells and reduces the degree of B cell immune response (73). However, the correlation between FAP and B cells in RA remains unclear, although we suspect some potential interactions, similar to other disease areas, require further exploration.

The immune response to citrulline protein is one of the typical pathological features of RA. A previous study confirmed that the immune process associated with citrulline protein influenced the degree of clinical symptoms in RA. Patients with undetectable levels of serum anti-citrullinated protein antibodies (ACPAs) outperformed serum ACPA^+^ patients in terms of joint swelling scores and blood sedimentation. Besides, ACPA expression can be found in synovial effusions of swollen joints even in serum ACPA^-^ RA patients (108). These results underscore the importance of the immune process at the local synovium in RA. The presence of citrullinated enzymes, including peptidyl arginine deiminase (PADI), and citrullinated proteins in the synovial tissue, and the presence of more citrullinated enzyme substrates on the surface of FLSs all make FLS the main location for this immune process. Although the possibility that FAP is involved by virtue of its unique enzymatic activity is worth investigating, clear experimental evidence is lacking so far. Moreover, an *in vitro* experiment in patients with RA demonstrated that a Th1 cell-activated microenvironment significantly enhances the activity of the major histocompatibility complex (MHC) class II shared epitope on the FSL surface (109). Meanwhile, studies have shown that a class of follicular helper T cells that need to be recognized by MHC class II in RA can promote inflammatory response (110). This series of processes increases the opportunity for FLSs to act as a reservoir for immunogenic molecules and to participate in autoimmune processes by assisting in the initiation or amplification of adaptive immune responses associated with RA (111). However, the involvement of FAP in this pathway remains elusive. Future studies should aim to explore more potential relationships between FAP and related citrulline proteins or other FLS surface proteins, which may improve our understanding of FAP-mediated immune function.

Such an immune profile also brings about an “immune-inflammatory” chain reaction. As mentioned earlier, the citrullinated proteins play an important part in RA, and related citrullinated autoantigens have been shown to promote the secretion of inflammatory factors in FLSs (112). In addition, the co-culture of FLSs with their recruited T cells produced a hyaluronic acid-rich synovial microenvironment in the ECM, which also significantly enhanced the expression of inflammatory cytokines, including IL-1, IL-6, IL-8, and TNF-α (68). FAP is expressed by FLSs in response to pathological conditions in the RA synovium, which is widely believed to act as a key regulator in the pathological microenvironment (113). Because of its potential association with T cells and its powerful immunomodulatory capacity, FAP may be involved in inflammation-related phenotypic changes in FLSs through multiple immune-related pathways. A previous study in vascular smooth muscle cells proposed that one of the disease characteristics of FAP^-^ female mice is a reduction in macrophages, which consequently reduces inflammation (76), demonstrating that the immune-inflammation pathway is an important modality of FAP regulation and providing ideas for RA-FAP-related research.

FAP expression performs regulatory functions in the interaction of FLSs with immune cells. Such cellular interactions are currently being studied extensively in the field of oncology and include suppression of self-reactive T cell proliferation and signal transduction, recruitment of Tregs and promotion of their differentiation (71), and acceleration of the release of cytokines such as TGF-β and CC motif chemokine ligand 2 (CCL-2) (74, 75), which positively promote feedback activation of high FAP expression (114). FAP has been confirmed to promote macrophage production in the tumor microenvironment and lead to an imbalance of M1/M2 macrophages (77), suggesting that FAP contributes to the recruitment and infiltration of other immune cells such as myeloid-derived suppressor cells (MDSCs) and neutrophils (115, 116). Unfortunately, these mechanisms have not been demonstrated in the RA synovial environment, highlighting an urgent need for research on cellular cross-linking in which FAP may intervene.

In summary, FAP can play an important role in initiating and maintaining abnormal adaptive immune responses by mediating the interaction of FLSs with immune cells, controlling the secretion of a variety of cytokines, and participating in the remodeling of the local immune environment. Due to the presence of abundant functional proteins on the surface of FLS, the absence of FAP in influenza has been reported to not overly affect immune function (117). A study in cancer-associated fibroblasts confirmed that FAP suppresses immune cell response by enhancing MDSC recruitment by promoting the STAT3 C-C motif chemokine ligand 2 signaling (118). Meanwhile, some studies have also indicated that FAP has immunosuppressive effects, and that CD4^+^/CD8^+^ T cell activity is increased after removing FAP^+^ cells in mice (119). These findings, which are outside the field of RA and have been reported inconsistently, highlight the importance of carefully considering whether FAP on the surface of FLSs is an indispensable player in the immunomodulatory function in RA and elucidating the role of FAP in immunomodulation.

### 4.2 Inflammatory responses

The inflammatory response demonstrated by FLSs plays an important role in the development of chronic inflammation in RA. When RA-susceptible individuals are exposed to stimuli such as environmental changes and injuries, the autoimmune response in the body is activated, resulting in the formation of an inflammatory microenvironment for RA and the accumulation of pro-inflammatory factors that cause continuous activation of FLS (120, 121). Activated RA-FLSs directly secrete inflammatory factors and chemokines that promote and maintain joint inflammation and retard its resolution. FAP contributes to the inflammation process. A clinical study on non-small cell lung cancer reported an increase in the peripheral neutrophil-to-lymphocyte ratio in patients with high FAP chromosome percentages (122). An *in vivo* study suggested that FAP deficiency in the synovial tissue of RA mice ameliorates inflammatory destruction of joints by histomorphometry (78). Both studies highlight the important role of FAP in the inflammatory response. Inflammatory responses mediated by immune pathways have been reported to play a major role in the inflammatory phenotype. Outside of these mechanisms, a number of physiological or pathological activities can cause inflammation to occur.

Endoplasmic reticulum stress (ERS) is a manifestation of cellular self-regulation. Unbalanced ERS in RA can exert pro-inflammatory properties by producing various relevant cytokines while enhancing the activation of FLSs by multiple toll-like receptor ligands (123), which has been shown to exacerbate the progression of inflammation in RA. Researchers studying primary human skin fibroblasts have shown that the activation of the structural domains in FAP is associated with upregulation of the ERS target phospho-eIF2α (79), and that overexpressed FAP may enhance the pro-inflammatory capacity of ERS in the process. Thus, an unidentified FLS-FAP-ERS pathway is likely to exist in the RA microenvironment. In addition, FAP expressed by pre-adipocytes has been shown to regulate signals such as the mammalian target of rapamycin (mTOR) by mediating ECM remodeling and to alter lipid metabolism (80), while FAP-mTOR-related signaling has been shown to alter collagen deposition in idiopathic pulmonary fibrosis (IPF) (81). Similar mechanisms in RA remain to be validated.

Using imaging analysis in patients with fibrotic disease and RA, existing clinical studies have identified a positive correlation between FAP expression and the degree of inflammatory lesions such as lymphoplasmacytic aggregation and joint destruction (124, 125). These findings support our belief that FAP is involved in the activation of the inflammatory phenotype from a molecular perspective. On this basis, the specific molecular mechanisms of FAP and RA-FLS inflammation-related signaling pathways are worthy of further investigation.

### 4.3 Outward invasion

As a member of the DPP family, FAP shows a specific structure and possesses a unique endopeptidase activity that is lacking in DPP-4 and can cleave peptide chains on its own (126). Collagen fibers—a major component of the extracellular matrix (ECM)—can be digested as a physiological substrate for FAP (127), which occurs after the cleavage of normal collagen fibers by matrix metalloproteinase-1 (MMP-1) secreted by FLS (82). Correspondingly, α2-antiplasmin (α2AP) in peripheral tissues has been described as a potential substrate for FAP. α2AP becomes a more potent fibrinolytic inhibitor after cleavage by FAP, leading to impaired fibrinolysis, which contributes to pathological fibrous lattice formation and accelerated fibrin deposition in the synovium (83, 84). These processes have been demonstrated in the field of oncology, and a similar response can be presumed to occur in the joint microenvironment of RA, with the digestion of ECM by FAP ultimately driving the peripheral invasion of FLSs. Although these fibrin-related pathological responses will ultimately contribute to the development of RA, the underlying mechanisms need to be understood in depth (128, 129). Maintenance of an activated state and recognizing specific substrates for cleavage and digestion is an important way for FAP to participate in the pathological ECM remodeling process through its biological enzymatic activity. This gives FLSs the most direct invasive power over the surrounding area.

Several studies have also demonstrated that FAP can activate relevant signals in the absence of enzyme catalytic activity (130), resulting in a stronger invasive capacity of cells (131). The ERK, nuclear factor kappa-B (NF-κB), and TGF-β signaling pathways are thought to be involved in the process of invasion along with FAP (132–134). FAP can synergize with cytokines such as integrin and urokinase plasminogen activator receptors. For example, FAP is activated by αvβ6-integrin during tumor progression, which contributes to the recruitment of relevant aggressive cytokines, including MMPs, in activated cancer-associated fibroblasts (38). In the synovial environment in RA, MMPs are important for cartilage degradation and act as markers of osteoclast expression, directly destroying local joint bone (135). On the basis of these findings related to FAP, we hypothesize that similar response pathways may exist in RA, which may be a new direction to explore FLS-induced bone erosion.

Studies of skin system disorders show that high expression of FAP directly or indirectly increases the invasive ability of keloid fibroblasts, and that selective inhibition of FAP leads to reduced invasion (136). However, the effect of FAP^+^ FLS invasion on bone destruction during RA progression is controversial. Previous studies have suggested an increase in bone destruction after FAP inhibition (137). This conclusion is not entirely convincing because the environment in which FAP expression occurred in these studies did not accurately match the synovial characteristics in RA patients (138). The more mainstream view is that FAP expression is strongly associated with increased bone destruction (139), and that FAP expression in synovial tissue is consistently higher in active RA patients than in patients in remission or normal adults (140, 141). Moreover, high levels of FAP mediate bone and cartilage destruction, in which members of signaling pathways such as Wnt have been shown to be an important regulator through the RA-FLS (142).

### 4.4 Cell migration

Symmetrical joint swelling is often observed in RA, with the progressive manifestation of adjacent joint spread and multi-system symptoms such as cardiac and pulmonary symptoms outside the joints in severe cases (143). Local migration of activated FLSs to healthy tissue outside the primary diseased joint is an integral part of such disease progression (144). We believe that the high expression of FAP also facilitates this process by acting synergistically with cytokines such as IL-1β and TGF-β. FAP positively stimulates the induction of differentiation of human bone marrow-derived MSCs to FLSs, reducing cell adhesion and inducing cell body contraction by inhibiting intracellular signals such as RhoA GTPase (85, 86) and thereby facilitating cell migration. Although the number of similar studies related to RA is currently low, studies of FAP in other disease areas, especially studies on cancer-associated fibroblasts, can provide a reference. Previous melanoma-associated studies have reported that cells showed greater migration ability after the induction of FAP expression in fibroblasts (145).

Moreover, in cancer-associated studies, FAP and DPP-4 have been shown to form heterodimers that form pseudopod-like complexes on the cell surface, providing conditions for subsequent cell migration (87). The association of FAP with β1-integrin has also been shown to play a key role in cell migration. The addition of an integrin inhibitor to FAP^+^ fibroblasts has been previously reported to reverse the migration phenotype (88). Meanwhile, FAP exhibits strong bioregulatory activity in the cross-linking of fibroblasts with other cells and contributes to the induction of other cells’ migration. For example, FAP^+^ fibroblasts in human breast tumor stroma can greatly increase cancer cell migration and induce epithelial-mesenchymal transition. This process is often accompanied by the activation of ERK and expression of MMP-1 (146). However, these FAP-mediated cellular activities have almost exclusively been demonstrated in oncology, and no concurrent evidence for the synovial microenvironment of RA has been reported to date. Cell migration involves complex physiological transduction mechanisms, and blocking mitogen-activated protein kinase (MAPK), phosphoinositide 3-kinase (PI3K)/serine-threonine protein kinase (AKT), NF-κB, and other signaling pathways is currently thought to inhibit the cell migration process associated with RA-FLS (147, 148).

In the synovial tissue of RA, immune cells often appear to infiltrate at a high level (149) and FLSs can be involved in the recruitment of immune cells such as Th17 (150), with various types of activated immune cells being attracted to FLSs and migrating toward the diseased synovial membrane (151). Immunomodulation is also involved in the FLS self-migration process. Migration of FLSs may be related to the increase in ACPA after synovial inflammatory injury, and the inhibition of citrulline enzyme binding to citrullinated proteins may help reduce the expression of ACPA and thus the migration of FLSs (152). As mentioned previously, FAP exhibits biological activity that is potentially associated with citrulline enzymes such as PADI, which may accelerate FLS migration. ACPA-containing IgG antibodies have been reported to stimulate the formation of neutrophil extracellular traps (NETs) by neutrophils recruited by RA-FLSs in a concentration-dependent manner (89). NETs are large DNA-based reticulation structures released by neutrophils that are highly activated in response to stimuli, and they have been extensively studied in oncology (153). In RA, NETs can induce FLS activation and enhance their migration and invasion abilities (90). FLSs continue to migrate and invade the cartilage lining and more distant skeletal joints (154), and this cellular activity resembles distant metastasis of cancer cells. Abundant NETs that can attract cancer cells have been shown to exist in target organs that are vulnerable to distant invasion by cancer cells. Cancer cells show specific receptors for NETs on their surfaces (155). Similar cellular responses may presumably occur in RA during joint involvement and the formation of extra-articular lesions, and FAP serves as one of the receptors on the surface of FLSs that may recognize each other with NETs and be a targeted therapeutic surface biomarker for blocking RA progression.

### 4.5 Synovial proliferation

In the RA synovial microenvironment, which is characterized by the accumulation of inflammatory factors such as TNF-α, the lining layer gradually transforms into a proliferative tissue structure with an invasive capacity (156). Activated FLSs maintain the proliferation and formation of the synovial layer almost exclusively as enlarged synovial layer cells, even dispersing in the synovial fluid to penetrate into the joint cavity (157). During the progression of RA, FLSs exhibit resistance to apoptosis (158). Overexpression of FAP has an important effect on the proliferative capacity of FLSs.

In addition to its role as a mediator in the immune regulation of FLSs, PADI intervenes in FLS proliferation and apoptosis. *In vivo* experiments have demonstrated that RA-FLSs increased PADI-4 expression under hypoxic conditions and significantly promoted the proliferation of FLSs through a feedback loop, while PADI-4 knockdown promoted FLS apoptosis (159). However, the relationship between FAP and PADI-4 levels is still unclear. An *in vitro* study in RA-FLSs found that FAP expression released the contact inhibition between cells and participated in cell cycle regulation through cytokines such as P53 and P21 (66). Meanwhile, several cancer-associated studies have shown that high expression of FAP promotes cell cycle progression, while FAP silencing is accompanied by cell cycle silencing (160, 161). FAP is an upstream regulator of the Rs-ERK signaling pathway and can serve as a proliferation marker for a range of cells, including lung adenocarcinoma and oral squamous carcinoma cells (162, 163).

The imbalance between survival expression factors and apoptosis inhibitory factors is also a cause of FLS overproliferation. RA-FLSs can be activated by the stimulation of cytokines such as IL-38, which induce the expression of pro-inflammatory cytokines (IL-6 and TNF-α) and angiogenic factors (vascular endothelial growth factor [VEGF] and HIF-1α) (164) that control RA-FLS proliferation and apoptosis in a concentration-dependent manner. Other studies have confirmed that IL-33 can also promote this process and that the NF-κB pathway may be involved (165). However, the role of FAP in this process remains to be investigated.

Contrary to its pro-inflammatory effect, ERS can also induce apoptosis in FLSs, providing microenvironmental protection in the arthritic synovium (166). Activated RA-FLSs show some resistance to ERS-induced apoptosis (123); this response may be attributed to differential protein expression on the FLS surface, which may reflect the double effect of FAP pro-inflammation and inhibition of apoptosis during ESR imbalance, but the exact mechanism remains poorly understood.

Several studies have shown that FAP is involved in the non-apoptotic death of other cells. For example, necrotic apoptosis of neutrophils in RA can be blocked by high expression of FAP when the enzymatic function of FAP plays a potential role, with the involvement of receptor-interacting protein kinase-3 (RIPK3) and mixed linage kinase-like (MLKL) (91). All these facts prove that FAP is important in cell growth and cell death processes such as proliferation and apoptosis. Under the influence of related mechanisms, growing FLSs and other cells further contribute to inflammation and cellular invasion through a feedback loop.

### 4.6 Local angiogenesis

Angiogenesis promotes pathological synovial development following the excessive local inflammatory response and immune imbalance (167). The generation of new blood vessels is regulated by angiogenesis-stimulating and angiogenesis-inhibiting factors, and a disturbed microenvironment can upset this balance. In the presence of local infiltration of immune cells with inflammatory factors and abnormal proliferation of synovial tissue, the new vascular system is generated at an accelerated rate to meet oxygen and nutrient requirements (92). A direct clinical link between poor patient prognosis and angiogenesis at the synovial site has been demonstrated in RA (168).

High expression of FAP in colorectal cancer is thought to promote angiogenesis (169). FAP^+^ cells enhanced abnormal angiogenesis by co-interaction with glioma cells (93), and FAP levels were positively correlated with angiogenesis in gastric cancer (170). Inhibition of FAP reduced the vascular density in epithelial tumors (171). The enzymatic activity of FAP may play a role in angiogenesis, for example, by binding growth factors such as neuropeptide Y (NPY) as substrates (172). NPY is a potent pro-angiogenic factor (173, 174), and the possibility that it can become more active after being catalyzed by FAP should be investigated in more detailed studies. Research in the field of oncology shows that FAP increased the expression of pro-angiogenic factors such as VEGF and decreased the level of anti-angiogenic molecules through paracrine communication (93), with the AKT and ERK signaling pathways being involved in this process (175, 176). A pro-angiogenic signaling molecule, matrix metalloproteinase-9 (MMP-9), is often co-expressed with FAP in corneal neovascularization, which may account for the angiogenetic phenotype (94). Activated FLSs in RA can express VEGF, angiopoietins, etc., as major players in the process of angiogenesis (95), and other matrix remodeling transcripts such as MMP-1 and TGF-β1 have been shown to be synchronously upregulated with FAP in dedifferentiated mature adipocytes (96). These studies suggest an inevitable link between FAP and local angiogenesis. However, the mechanism underlying their cross-linked expression in RA has not yet been defined.

Current research in many disease areas has linked FAP to angiogenesis in the microenvironment, which may provide insights into understanding the generation of local abnormal synovial vessels (vascular opacities) in RA.

## 5 Therapeutic strategies targeting FAP

Although several treatments for RA are available, patients still show a persistent inflammatory state and experience a high risk of physical disability (177). Systemic anti-inflammatory therapy has been reported to improve targeting synovitis in patients (178). Infiltration of the local synovial membrane by multiple inflammatory and immune cells results in increased secretion of large amounts of inflammatory factors and chemokines, which cause cartilage erosion. The existing biologically targeted agents aim at key cytokines, mainly TNF and the IL-6 family (179, 180), and have been reported to reduce disease activity and achieve partial remission of RA, but they may cause undesired immune suppression. Therefore, more specific targeted therapeutic modalities are needed and activated FLS surface biomarkers should be identified for targeted therapy (156). Several clinical trials have been performed to evaluate the clinical value of molecular markers such as CDH-11 and CD90 (181, 182), but no significant targeted therapeutic effect have been found to date. In fact, using FAP as a target and killing FLSs selectively has been tested in synovial membrane *ex vivo*, and several studies have demonstrated that this is a viable way forward (8, 65).

Although FAP can be detected in tissues, tissue-based evaluations do not seem to be an effective quantitative measurement. Using the reaction rate of substrates such as 3144-aminomethylcoumarin and fibroblast growth factor 21 to calculate the FAP level is one approach (183, 184), but the accuracy of such measurements may be affected by the reaction microenvironment and the presence of other enzymes. These limitations indicate the need to identify a highly selective substrate for FAP (185), which can be applied to the preparation of clinical drugs that act through the binding of the enzyme to the substrate specifically.

Another viable strategy is to develop highly selective inhibitors of FAP enzyme activity. However, due to the similarity of FAP to members of the DPP family, identification of a suitable specific inhibitor is a significant challenge. A previous study proposed that the compound Ac-Gly-BoroPro may act as an inhibitor of FAP with 7-fold more selectivity than other DPPs (186). Another novel inhibitor, IOCB22-AP446 (6d, IC_50_ = 89 pM), was reported to be 36-fold more potent than other effective inhibitors reported to date (187). However, most inhibitors will still act in combination with DPP-4, which has the highest similarity to FAP (188). Although FAP inhibitors have great therapeutic potential, their effects on RA are still unknown. Luna Ge et al. found that the FAP inhibitor [^18^F] AlF-NOTA-FAPI-04 was an effective radiotracer with high uptake in the arthritic synovium and was suitable for RA-FLS imaging. Thus, this radiotracer can be applied for disease identification and diagnosis (189). However, inhibitors only block FAP activity and do not cause harm to FAP^+^ cells; therefore, when used in RA-FLS, the effect of such drugs may not be satisfactory because other proteins such as CD90 and CD34 are still expressed on the surface of FLS. The application of FAP inhibitors in the clinical treatment of RA has not been sufficiently studied, indicating the need for additional studies to verify whether they are effective and safe.

A study on cardiac fibrosis reported that modified T cells that express chimeric antigen receptors (CARs) against disease-associated antigens can target FAP to clear activated cardiac fibroblasts (190). This approach has been approved for use in hematologic malignancies (191), but conventional CAR-T cells are not very stable and often cause off-target effects. Because CAR-T cells can remain in the body for a long time, they cause a continuous release of cytokines, which may easily induce damage to other tissues, and the resultant side effects are difficult to prevent (192). In recent years this approach has been tried in the treatment of RA with good experimental results (193), but since it is not yet clinically applicable and no investigator has linked it to FAP-targeted therapy, its pros and cons still need to be evaluated.

Another approach for tumor treatment involves the activation of combinations called “prodrugs” by FAP (194). The prodrug consists of a therapeutic cytotoxic agent composed of a polypeptide chain that can be hydrolyzed by FAP. The action of the prodrug is limited by the structural features, and upon entry into an environment with high FAP expression, the polypeptide chain is specifically hydrolyzed to release the cytotoxic drug it carries. The prodrug uses the enzymatic action of FAP to kill FAP^+^ cells, a mechanism more often used in tumor therapy to help the drug unlock its target more precisely and avoid damage to normal tissue from non-specific cytotoxic drugs (195). To treat RA, drugs that can achieve targeted therapy against synovial tissue and maintain stable blood concentrations should be designed.

The physiological characteristics of FAP, which is barely expressed in normal tissues and is exposed to the cell surface as a membrane protein, allow researchers to use it as a potential target for treatment (196). To develop targeted FAP for the treatment of RA, researchers should determine whether it is important to kill FAP^+^ FLSs or to inhibit FAP activity. The results of surface molecular identification of local tissue cells in the synovium can suggest the relative amount of FAP^+^ FLSs, which will facilitate selection of the appropriate approach.

## 6 Conclusion

The presence of FAP^+^ FLSs in RA synovial tissue often predicts poor clinical outcomes. FLSs play a key role in RA by regulating various cellular phenotypes throughout the course of the disease. Thus, understanding the pathological changes in FLSs may help develop new therapeutic approaches for the treatment of RA. FAP is currently one of the prominent markers that has received much attention, but most of the research on FAP has been conducted in oncological diseases, and its significance in RA has not been explored in depth. In this article, we summarize the role and mechanisms of FAP-mediated FLSs in the pathogenesis of RA on the basis of the available evidence, inevitably drawing on some research advances in other disease areas in the process. Under these conditions, it is important to carefully consider whether FLSs play a similar role in different diseases and whether changes in the microenvironment have a differential impact on FAP. This aspect needs to be explored in depth by more studies. The unique activity of FAP allows it to confer specific biological functions to FLSs. This property can be used to study the synovial joint microenvironmental changes induced by FAP expression. However, there remain many unanswered questions regarding the detailed molecular mechanisms of interaction between FAP and FLSs. Further in-depth studies are advocated to clarify these unanswered questions in the RA field.

## Author contributions

All authors contributed to the general design of the study. YX conceptualized the review. ZW drafted the review and JW prepared the figures. All authors reviewed and revised the manuscript. All authors agreed to take responsibility for all aspects of the work. All authors contributed to the article and approved the submitted version.
